# Structure‐Based Screening of Tetrazolylhydrazide Inhibitors versus KDM4 Histone Demethylases

**DOI:** 10.1002/cmdc.201900441

**Published:** 2019-10-10

**Authors:** Piotr H. Małecki, Nicole Rüger, Martin Roatsch, Oxana Krylova, Andreas Link, Manfred Jung, Udo Heinemann, Manfred S. Weiss

**Affiliations:** ^1^ Macromolecular Crystallography Helmholtz-Zentrum Berlin für Materialien und Energie Albert-Einstein-Str. 15 12489 Berlin Germany; ^2^ Macromolecular Structure and Interaction Max Delbrück Center for Molecular Medicine Robert-Rössle-Str. 10 13125 Berlin Germany; ^3^ Institute of Pharmacy Universität Greifswald Friedrich-Ludwig-Jahn-Str. 17 17489 Greifswald Germany; ^4^ Institute of Pharmaceutical Sciences University of Freiburg Albertstr. 25 79104 Freiburg Germany; ^5^ Department of Molecular Biophysics Forschungsinstitut für Molekulare Pharmakologie Robert-Rössle-Str. 10 13125 Berlin Germany; ^6^ Current address: International Institute of Molecular and Cell Biology Ks. Trojdena Street 4 02-109 Warsaw Poland; ^7^ Current address: Københavns Universitet Center for Biopharmaceuticals Universitetsparken 2 2100 Copenhagen Denmark

**Keywords:** cancer, drug discovery, inhibitors, proteins, structure elucidation

## Abstract

Human histone demethylases are known to play an important role in the development of several tumor types. Consequently, they have emerged as important medical targets for the treatment of human cancer. Herein, structural studies on tetrazolylhydrazide inhibitors as a new scaffold for a certain class of histone demethylases, the JmjC proteins, are reported. A series of compounds are structurally described and their respective binding modes to the KDM4D protein, which serves as a high‐resolution model to represent the KDM4 subfamily in crystallographic studies, are examined. Similar to previously reported inhibitors, the compounds described herein are competitors for the natural KDM4 cofactor, 2‐oxoglutarate. The tetrazolylhydrazide scaffold fills an important gap in KDM4 inhibition and newly described, detailed interactions of inhibitor moieties pave the way to the development of compounds with high target‐binding affinity and increased membrane permeability, at the same time.

## Graphical abstract

**Bring on the subs**! Tetrazolylhydrazide compounds are isosteres of 2‐oxoglutarate, which is the natural cofactor of KDM4 proteins. It is believed that substitution of the carboxylic groups with tetrazole and hydrazide moieties will lead to KDM4 inhibitors with improved physicochemical properties. The binding mode of various such ligands in the active site of KDM4D is elucidated and movements of the interacting amino acid residues upon ligand binding are described.

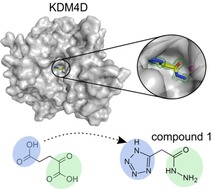

## Introduction

Gene expression regulation through epigenetic factors is a concept that is well established nowadays. It is defined as a pattern of changes in gene expression without altering the underlying DNA sequence.^1^ Living cells must be able to dynamically respond to changes in physiological and environmental stimuli and to modify their chromatin structure accordingly. A complex set of regulatory events, for example, DNA modification by cytosine methylation or covalent histone modifications have been shown to alter the interactions between DNA and histones. Modifications promote either compaction or relaxation of the chromatin, and thus, play a direct role in controlling the transcription level of particular genes. Epigenetic control occurs in both healthy and cancerous cells.^2^ For instance, aberrant histone modification exhibits a clear relationship with disease and is often associated with cancer.^3^ The ability to interfere with these processes is therefore of increasing interest for researchers from the academic sector and from pharmaceutical companies.

One of the most studied histone modifications is the addition of a methyl group to lysine residues, as a result of the action of histone lysine methyltransferase enzymes (KMTs).^4^ The histone tails, which are packed more loosely than the histone cores, have been identified as the main targets for modifications by KMTs.^5^ Despite the subtle physicochemical effect of lysine methylation, it has a tremendous regulatory impact on cellular functions. Combining the number of lysine residues in histones, which can be methylated, the various degrees of methylation (mono‐, di‐, and trimethylation), and the fact that methylation is cooperative with other modifications results in a very complex picture for gene expression control.^6^ Until about 2004, histone lysine methylation was believed to be irreversible. Since then, two classes of reverse‐action (eraser) enzymes—histone lysine demethylases—have been discovered and studied in depth. Usually, these enzymes are selective for a particular lysine residue in a particular methylated state. The first class comprises the flavin‐dependent lysine‐specific demethylases (LSDs or KDM1s). The second class, which has more members than the first, comprises all oxygen‐dependent demethylases, which use iron(II) and 2‐oxoglutarate (2OG, also called α‐ketoglutarate) as cofactors. The enzymes, which belong to the second class, contain the Jumonji domains; hence, they are also called JmjC KDMs.^7^ The discovery of these enzymes established an entirely new concept, with respect to the dynamic reversible regulation of histone methylation by KMTs and KDMs.

In humans, the JmjC histone lysine demethylases encompass distinct families, each of which consist of several multidomain members. There are at least six KDM families recognized in the literature (KDM2–7) and still new subfamilies (KDM8 and KDM9) are being reported. The catalytic function of these enzymes is provided by the JmjC domain, whereas the JmjN domain closely interacts with JmjC and provides the three‐dimensional scaffold. All members are classified based on the presence of conserved structural elements, in addition to the catalytic JmjC domain, including PHD, Tudor, CXXC, FBOX, ARID, LRR, and JmjN domains.^7^, ^8^

The five members of the KDM4 subfamily (KDM4A–E) catalyze the removal of the methyl group from tri‐ and dimethylated lysine residue 9 in histone 3 (H3K9me3/me2). At the same time, KDM4A–C also process the tri‐ and dimethylated lysine residue 36 (H3K36me3/me2). Increasing evidence has been accumulated that KDMs are also linked with various disease states.^9^ For instance, KDM4B was found to be involved in breast, colon, and gastric cancer,^10^ whereas KDM4A, KDM4C, and KDM4D are overexpressed in prostate cancer, during which they act as androgen receptor coactivators.^11^ Consequently, these enzymes have been identified as potential targets for intervention by drugs. At present, four compounds targeting LSD1 are in clinical trials, whereas for the JmjC KDM family of enzymes no such developments have been reported.^12^

In spite of this, a large number of small‐molecule inhibitors of JmjC KDMs have been established. For the KDM4 subfamily alone, about 94 structures with bound ligands^13^ have been deposited in the Protein Data Bank (PDB).^14^ Most of the inhibitors are 2OG competitors that coordinate the Fe^2+^ ion in the catalytic center and sometimes penetrate into the histone peptide binding pocket. Many of these compounds mimic the natural cofactor and, similar to 2OG, possess a carboxylic acid moiety. The carboxylic group is a common building block in endogenous substances, but it is also part of many pharmacophores.^15^ Its success is due to its acidic character and potential to engage in relatively strong electrostatic interactions and hydrogen bonds. On the other hand, the carboxylic moiety reduces the ability of a drug to passively move across biological membranes, and thus, limits cell permeability. Two different strategies are suited to overcoming this problem associated with drugs and drug candidates: 1) bioisosteric replacement or 2) prodrug strategies, such as use of carboxylic acid esters. Consequently, in a drug development process of carboxylic acid leads against JmjC KDMs, several ester compounds have been investigated.^16^ Because this well‐established strategy potentially yields prodrugs that are transformed into the active form differently in individual patients, the replacement of the carboxylic acid by groups with similar volume, shape, and physicochemical properties is an attractive alternative. From this perspective, it is quite surprising that structural data for KDM4 proteins and compounds containing tetrazole moieties have not been reported, although the tetrazole moiety is part of many biologically active compounds, including drugs.^17^ Although the electrostatic potentials of carboxyl and tetrazole groups are similar, tetrazoles could improve the cellular permeability due to their more lipophilic character. Tetrazoles are low‐toxic, metabolically stable bioisosteres of carboxylic acids, and thus, promising building blocks for analogues of the natural KDM4 cofactor, 2OG.^18^ However, none of the KDM4–ligand complexes reported so far in the PDB contain a tetrazole ring as a functional group.

In 2015, Rüger et al. identified tetrazolylhydrazides as selective fragment‐like inhibitors of the KDM4 subfamily of proteins.^19^ Arrays of such molecules have since been synthesized and analyzed mainly by molecular modeling approaches.^20^ The most promising compounds suggested a mode of binding in the KDM4 active site. The assumption was that the tetrazole group acted through binding to the 2OG site as a competitor for the substrate. However, direct support for this hypothesis by experimental structural analysis has so far been lacking.

**Figure 1:**
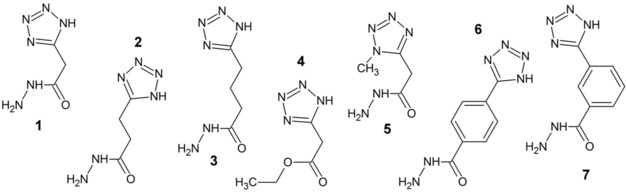
Structures of compounds **1** to **7**, which have been cocrystallized with KDM4D.

Herein, we present the first successful attempt to obtain high‐resolution X‐ray structures of both hydrazide and tetrazole moieties bound to the enzyme KDM4D. These small molecules constitute promising frameworks for early drug discovery stages because their initial selectivity has been reported.^19^ The structures presented herein show the specific binding of tetrazolylhydrazide ligands^19^, ^20^ (Figure 1) to the metal ion in the active site, serving as 2OG competitors. A series of molecules with hydrazide and tetrazole ring modifications show different modes of binding in the active center of the enzyme. Mapping of the active site allowed us to localize the residues that were able to adjust their position upon ligand binding. Our attempt to describe compounds that exclusively inhibit this relatively new target KDM4 has resulted in structural data that show the mode of binding of new functional groups that have not been described previously: tetrazole and hydrazide. Uncovering and describing these details is a step forward in the development of drug‐like molecules in the fight against cancer.

## Results

### KDM4D crystals and KDM4D structure

**Figure 2:**
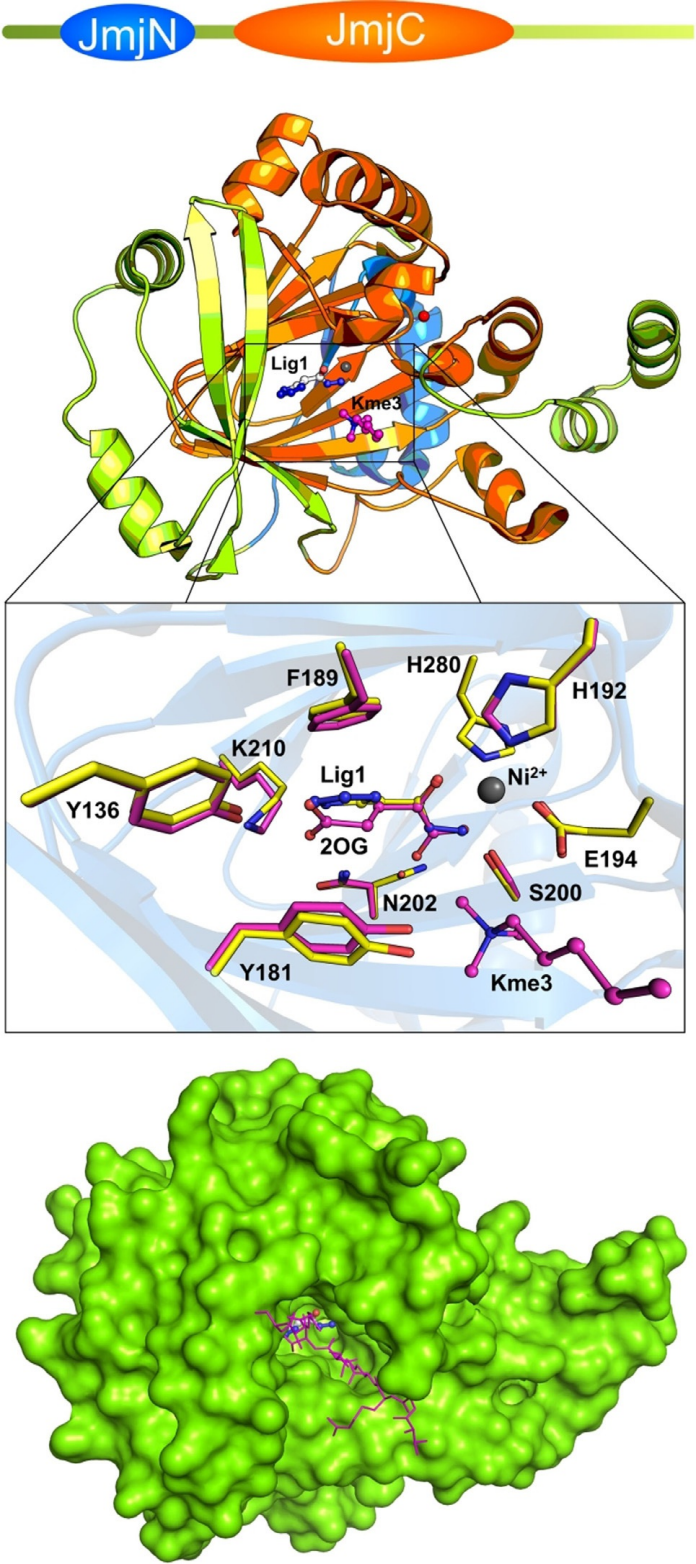
Structure and ligand binding of demethylase KDM4D. Top: Domain organization in KDM4D. The colors are in accordance with the secondary structure representation. Middle: The core domain of KDM4D in ribbon representation. The JmjN domain is colored in blue and the JmjC domain in orange. Ligand **1** structure and superimposed elements from the reported structure (PDB ID: https://www.rcsb.org/structure/4HON ^[13m]^)—cofactor 2OG and the “incoming” trimethylated lysine (Kme3, part of the histone like peptide)—can be seen in the active‐site pocket. Superposition of ligand **1** with the 2OG‐bound structure shows high structural similarities between bioisosteres. Substrate binding site residues with semitransparent secondary structure elements can be visible. The cofactor and trimethylated lysine residue are given in ball‐and‐stick representation in magenta, whereas the tetrazolehydrazide ligand and binding residues are in yellow. Bottom: Surface representation of KDM4D with the ligand in the binding pocket and the histone‐like peptide bound on the surface is superimposed in magenta as stick representation.

All structural work reported herein was performed on the catalytic core of KDM4D, which comprised amino acid residues 1–342. This construct crystallizes in a form that renders the active site of the protein fully accessible, with no obstructions from neighboring protein molecules in the crystal lattice. In all KDM4 subfamily members, the 2OG binding site is literally the same and engages the same residues. The tetragonal crystal form features one molecule in the asymmetric unit and permits the diffusion of ligand molecules through the solvent channels to the substrate binding pocket of the protein. This crystal form of KDM4D has been observed previously (PDB ID: https://www.rcsb.org/structure/3DXU, unpublished), and based on our own observations, it turned out to be superior to others for ligand soaking experiments due to its diffraction properties, mechanical stability, and tolerance for high DMSO concentrations. In all obtained structures, the part of the amino acid sequence that was visible in the electron density included residues 11–342. Overall, the structure of KDM4D is globular and may be divided into the JmjN and JmjC domains. The N‐terminal JmjN domain comprises residues 18–60 and the catalytic JmjC domain comprises residues 146–312 (Figure 2). These domains are highly similar to the homologous domains of other KDM4 proteins. As in other KDM4 subfamily members, a conserved zinc‐binding site composed by the residues His244, Cys238, Cys310, and Cys312 was observed. This binding site is of mainly structural importance. The Zn^2+^ ion is present with 100 % occupancy and has its origin in the *Escherichia coli* host organism because no Zn^2+^ was added to any of the purification or crystallization buffers. The active‐site metal center contains Ni^2+^ as the metal ion. Although in the natural form of the enzyme this site is occupied by an Fe^2+^ ion, it is widely accepted in crystallographic studies to replace rather oxygen‐sensitive Fe^2+^ with Ni^2+^ or Co^2+^. All of the ligands (**1**–**7**; Figure 1) reported in this study occupy the native cofactor binding site, which is in close vicinity to the metal binding site and the site binding the methylated histone lysine. Based on available statistics (Table 2) and the quality of experimental data, the structures reported herein are of sufficiently high quality to ascertain the binding of the soaked‐in ligands. The entire catalytic core and the binding of the cofactor 2OG and a trimethylated peptide that mimics the histone 3 tail (H3K9me3) has been thoroughly described by Krishnan and Trievel.13m Cofactor 2OG chelates the active‐site Ni^2+^ ion by using both the C2 keto group and C1 carboxylate group. In addition, the octahedral coordination sphere of the metal center contains Gln194, which binds opposite to the C2 keto group; His192, which binds opposite to the C1 carboxylate group; His280; and a water molecule. The other end of cofactor 2OG is held in place by Asn202, Lys210, and Tyr136 (Figure 2). The active‐site residues Tyr181, Glu194, and Gly174 are in close vicinity around the trimethylated lysine of the histone.13m The peptidic ligand was not used in our experiments; thus, it is not observed in the structures reported herein, but superimposed in Figure 2 for visualization of the histone binding site in KDM4 proteins.

### Ligand binding examined by crystal structure analysis

**Figure 3:**
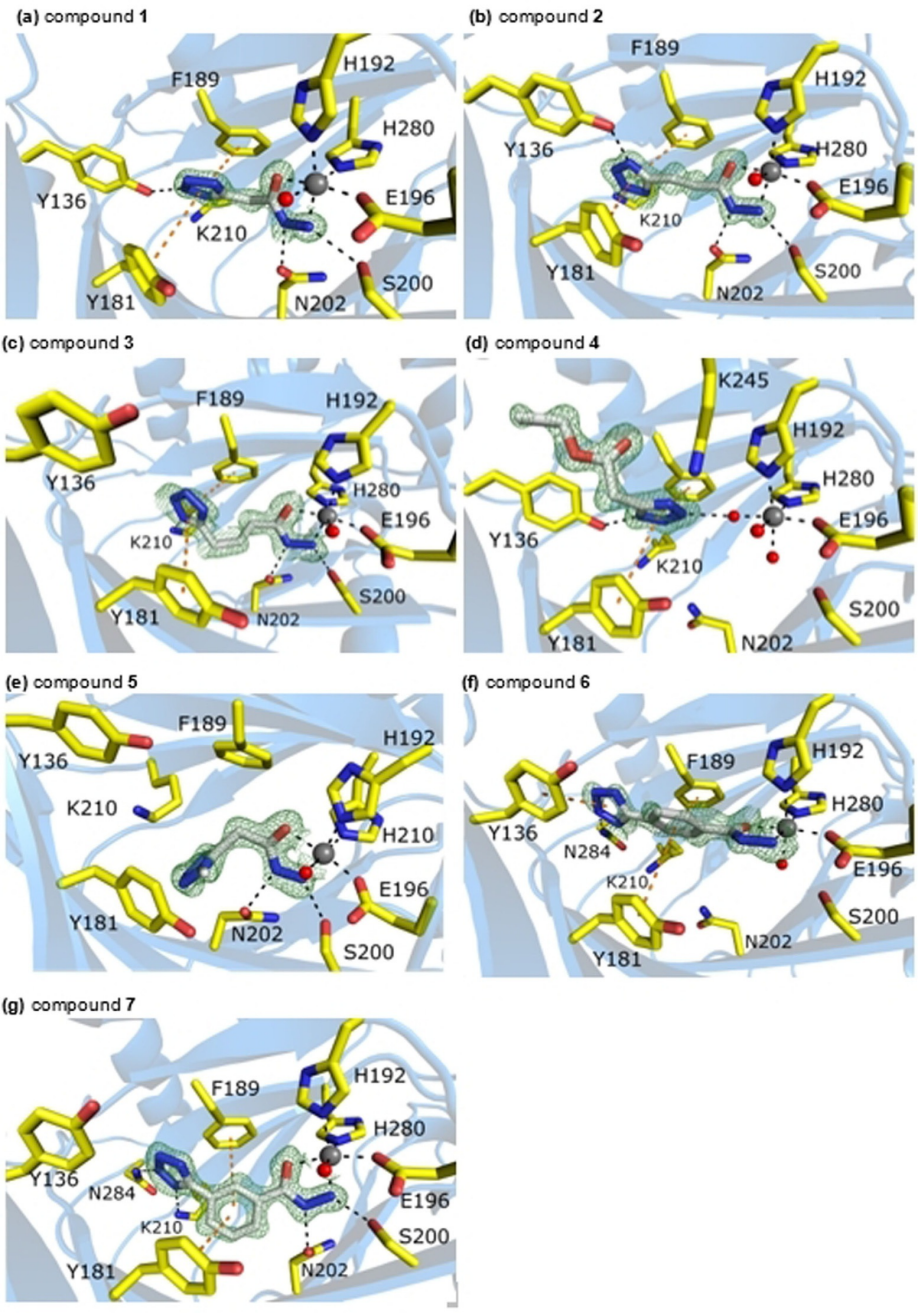
Details of the interaction of the seven ligands with KDM4D. The protein part of the structure is depicted as ribbons, except for the side chains of the relevant amino acid residues, which are shown as sticks. The presence of the ligand, also depicted as sticks, is evidenced by the (2mFo‐DFc, αc)‐composite omit map in green contoured at 1.5 σ for compounds **1** (A), **2** (B), **4** (D), **5** (E), and **7** (G); at 1.1 σ for compound **3** (C); and at 0.8 σ for compound **6** (F). Water molecules are shown as red spheres and the metal ion as a gray sphere. Polar interactions between the ligand and the protein, water molecules, and metal ion are indicated as black dashed lines. The orange dashed lines highlight aromatic stacking interactions.

Compounds **1**–**7**, which all contain a tetrazole group (Figure 1), were individually soaked into KDM4D crystals. This resulted in a series of seven crystal structures of KDM4D ligand complexes (Figure 3). The full picture of spatial positioning and detailed web of interactions of protein residues with compounds are discussed in the following subsections. All structures are of high quality, as evidenced by their resolution and refinement statistics (Tables 1 and 2). Although some ligands exhibit less than 100 % occupancy, which means that they are only bound to a fraction of the protein molecules, their clear appearance in the difference electron density map allows their unambiguous placement in the structure. All compounds in this series, except for compounds **4** and **5**, are composed of two building blocks intended as interaction motifs: the tetrazole ring and the hydrazide group. Ligands mainly differ in the alternations and modifications incorporated between them. The functional groups of the compounds were designed with binding to the KDM4 proteins through these two functional groups in mind. In addition to compounds **1**–**5**, which exhibit simple binding motifs and some modifications, two more complicated tetrazolylbenzohydrazide compounds (compounds **6** and **7**) were investigated. In these compounds, the tetrazole ring is attached in *meta* and *para* positions to the benzene ring.

**Table 1:** Data collection and processing statistics. The values in parentheses are for the respective highest resolution shell.

	**1**	**2**	**3**	**4**	**5**	**6**	**7**
beamline	BL14.3	BL14.1	BL14.1	BL14.3	BL14.1	BL14.1	BL14.1
*λ* [Å]	0.8943	0.9184	0.9184	0.8943	0.9184	0.9184	0.9184
crystal–detector distance [mm]	120.1	180.8	210.0	120.1	196.9	196.7	180.6
resolution range [Å]	48.01–1.33 (1.41–1.33)	47.99–1.18 (1.22–1.18)	48.04–1.35 (1.43–1.35)	47.9–1.26 (1.33–1.26)	47.92–1.47 (1.56–1.47)	48.17–1.28 (1.36–1.28)	48.24–1.33 (1.38–1.33)
space group	*P*4_3_2_1_2	*P*4_3_2_1_2	*P*4_3_2_1_2	*P*4_3_2_1_2	*P*4_3_2_1_2	*P*4_3_2_1_2	*P*4_3_2_1_2
unit cell *a*, *c* [Å]	71.63, 150.77	71.49, 150.44	71.67, 150.66	71.45, 150.54	71.46, 151.07	71.85, 151.53	72.00, 150.87
total no. reflections	687 453 (108 567)	1 671 018 (255 958)	1 122 708 (179 953)	1 069 690 (167 899)	587 371 (93 159)	890 260 (143 199)	1 187 218 (177 815)
no. unique reflections	168 562 (27 166)	247 119 (39 529)	165 316 (26 447)	202 280 (32 624)	127 491 (20 523)	195540 (31550)	174 441 (28 048)
multiplicity	4.1	6.8	6.8	5.3	4.6	4.6	6.8
completeness [%]	98.5 (98.6)	99.8 (98.7)	99.6 (98.3)	99.9 (99.6)	99.8 (99.3)	99.8 (99.3)	99.7 (99.2)
mean *I*/*σ*(*I*)	15.4 (1.8)	16.0 (2.0)	16.4 (1.8)	16.5 (2.0)	18.2 (2.0)	16.9 (1.9)	20.1 (2.4)
*R*_meas_ [%]	6.7 (86.9)	7.2 (95.5)	7.7 (103.4)	6.7 (92.9)	4.9 (78.7)	5.5 (85.3)	5.9 (79.9)
CC_1/2_	0.999 (0.613)	0.999 (0.694)	0.999 (0.676)	0.999 (0.679)	0.999 (0.685)	0.999 (0.636)	1.0 (0.77)
Wilson *B* factor (Å^2^)	11.5	11.0	13.5	11.3	18.1	13.0	13.0

**Table 2:** Refinement and validation statistics.

	**1**	**2**	**3**	**4**	**5**	**6**	**7**
refinement program	Refmac5 and Phenix.refine						
resolution	48.01–1.33	47.92–1.18	48.04–1.35	47.9–1.26	47.92–1.47	48.17–1.28	48.24–1.33
reflections in working set	166 462	244 532	163 219	200 134	125 406	193 437	172 341
reflections in test set	2086	2582	2090	2117	2081	2099	2117
*R*_work_ [%]	12.9	12.1	12.8	12.3	12.8	12.4	12.3
*R*_free_ [%]	15.6	15.0	15.9	14.6	15.3	15.8	15.4
no. non‐H atoms	3498	3646	3488	3530	3329	3495	3583
macromolecule	2957	3073	2970	2951	2914	2948	3043
ligands	65	79	47	78	52	70	44
water	459	474	452	486	351	464	478
RMS (bonds) [Å]	0.035	0.024	0.028	0.013	0.031	0.009	0.028
RMS (angles) [°]	2.6	2.2	2.3	1.6	2.4	1.4	2.3
Ramachandran favored [%]	99	99	99	99	98	99	99
clashscore	7.12	5.50	7.13	1.98	4.92	3.66	9.69
average *B* factor [Å^2^]	17.6	17.5	20.0	18.4	25.5	20.6	20.1
macromolecule	15.0	14.8	17.6	15.5	23.1	17.8	17.5
ligands	28.7	26.5	34.2	30.2	43.2	35.2	25.7
water	31.9	32.3	33.7	33.5	41.5	35.6	35.6
ligand occupancy	0.77	0.84	0.86	1	0.9	0.64	0.77
PDB ID	https://www.rcsb.org/structure/6ETS	https://www.rcsb.org/structure/6ETV	https://www.rcsb.org/structure/6ETW	https://www.rcsb.org/structure/6ETT	https://www.rcsb.org/structure/6ETE	https://www.rcsb.org/structure/6ETG	https://www.rcsb.org/structure/6ETU

### Structure of KDM4D with compound 1

The lead structure, compound **1**, represents the molecule with the shortest distance between the two interaction motifs: only one methylene group separates the tetrazole group and the hydrazide (*n*=1). In the structure of compound **1** bound to KDM4D, a clear positive difference electron density for the ligand could be observed and the ligand could be modeled with 77 % occupancy (Figure 3 A). Its hydrazide group chelates to the active site Ni^2+^, which is a surrogate of iron, as discussed above. The mode of chelation is very similar to that observed in the binding of the natural 2OG cofactor (Figure 2).13m The aromatic tetrazole ring is sandwiched between the two active‐site aromatic residues Phe189 and Tyr181. The distances between the tetrazole and the centers of the aromatic rings are 4.2 and 4.9 Å for Phe189 and Tyr181, respectively; thus indicating an aromatic stacking interaction. The distance of 2.6 Å between the hydroxy of Tyr136 and the second nitrogen atom from the tetrazole ring indicates strong hydrogen bonding. All of the other nitrogen atoms in the tetrazole ring are connected to the protein residues through clearly defined water molecules.

### Structure of KDM4D with compound 2

Compound **2** is characterized by two methylene groups between the functional groups (*n*=2). In the complex structure with KDM4D, this leads to a shift of the tetrazole ring relative to its position in the complex with compound **1**, whereas the hydrazide group remains unchanged (Figure 3 B). The observed electron density for the side chain of Tyr136 reveals that its position has changed. In its major conformation (64 % occupancy), the side chain has moved by 3.6 Å relative to its position in the structure with shorter compound **1**. The first nitrogen atom of the tetrazole ring interacts with Tyr136‐OH at a distance of 2.7 Å and the third nitrogen atom with Lys210‐NZ at a distance of 2.8 Å. The aromatic stacking interactions are still present, with distances between the centers of the aromatic rings of 4.3 and 4.6 Å for Phe189 and Tyr181, respectively.

### Structure of KDM4D with compound 3

In the case of an even longer ligand with *n*=3 (**3**), the hydrazide group remains in the same position as those reported for both *n*=1 and 2. The tetrazole ring still interacts with Lys210‐NZ, but it is shifted by about 1.8 Å away from the position observed in the case of compound **1**. It is now located at the position of Tyr136, and thus, forces the tyrosine aromatic ring further away, so that the residue–ligand interaction is no longer observed (Figure 3 C).

### Structure of KDM4D with compound 4

Compound **4** is characterized by the substitution of the hydrazide with an ester group. It could be modeled with clear electron density in the binding groove. Its tetrazole group is coordinated by two residues, Lys245 and Tyr136. Lys245‐NZ, the electron density of which usually identifies two distinct conformations, is clearly defined and pointing inward into the active site (Figure 3 D). The two remaining nitrogen atoms of the tetrazole are hydrogen bonded to water molecules and through one water molecule to the active‐site metal ion. The orientation of the ligand is flipped by 180° around the tetrazole ring relative to the orientation observed for compounds **1** and **2**. The position of the tetrazole ring is 1.4 Å away from the tetrazole in the case of ligand **1**. The ester tail of the ligand spans the binding cavity at its entrance, close to the surface of the protein, forming hydrophobic interactions with the protein.

### Structure of KDM4D with compound 5

Compound **5** is a variant of compound **1** with a methyl substituent at position 1 of the heterocyclic ring. The hydrazide group of the ligand is placed in the same position as that in all structures described so far and it chelates the active‐site metal ion. However, the whole decorated tetrazole group is turned by 85°, and its carbon atom points toward Tyr181, whereas the ring is placed between Asn202 and Lys210 (Figure 3 E). The third nitrogen atom of the ring binds to a water molecule through a hydrogen bond.

### Structure of KDM4D with compound 6

Compound **6** has a tetrazole ring in the *para* position of the benzene ring. The benzene ring is placed 4.4 Å away from Phe189 and 5.4 Å away from Tyr181. The second nitrogen atom of the tetrazole ring is hydrogen bonded to Asn284‐N and is oriented parallel to Tyr136 (Figure 3 F). Compared with all compounds analyzed previously, the hydrazide group is rotated by 90°. The nitrogen atom adjacent to the carbonyl group occupies the position of a water molecule, which is bound to the metal ion in previous cases, while the water molecule is now modeled in a position occupied by the nitrogen atom of the hydrazide group in other cases.

### Structure of KDM4D with compound 7

Compound **7** carries the tetrazole ring in the *meta* position of the benzene ring. The hydrazide group binds in the same way as that observed in all other structures (Figure 3 G). The benzene ring is positioned between both functional groups and is engaged in stacking interactions with Tyr161 and Phe189 at distances of 4.3 and 5.1 Å, respectively. The first tetrazole nitrogen atom hydrogen bonds with Lys210 (2.9 Å) and the second nitrogen atom with Asn284 (3.1 Å). The position of the ring is only 0.8 Å further away compared with the most similar ligand, compound **3**. Also, the aliphatic chain that links the tetrazole and hydrazide groups in compound **3** ligand is found at the same position as that of the benzene group of this ligand.

### Ligand binding investigation

#### Isothermal titration calorimetry (ITC)

The affinity and energetics of the binding of tetrazole ligands to KDM4D were studied by means of ITC. In all cases, the ligand was titrated into the solution of protein in the calorimeter cell. The binding of compound **1** to KDM4D is exothermic and characterized by a dissociation constant, *K*_d_, of 2.3 μm. Although the interaction is favored by both enthalpic (Δ*H*=−2.6 kcal mol^−1^) and entropic (−*T*Δ*S*=−4.9 kcal mol^−1^) contributions, the entropic part dominates and has a nearly twofold higher impact on the Gibbs free energy of binding (Δ*G*=−7.5 kcal mol^−1^; Table 3). Compound **2** displayed a rather large differential power signal, but this signal could not be attributed to binding. It most likely originated from side effects of the system, such as ligand dilution. No binding isotherms could be observed for compounds **3**–**6**. The binding of compound **7** to KDM4D was found to be endothermic. With *K*_d_=41 μm, it is weaker, relative to the binding of compound **1**. This interaction is characterized by an unfavorable positive binding enthalpy (Δ*H*=4.0 kcal mol^−1^), which is overcompensated for by the rather strong entropic contribution (−*T*Δ*S*=−9.9 kcal mol^−1^).

**Table 3:** Summary of ITC measurement binding parameters with errors.^[a]^

Ligand	*K*_d_ [μm]	Δ*H* [kcal mol^−1^]	−*T*Δ*S* [kcal mol^−1^]	Δ*G* [kcal mol^−1^]	*N*
**1**	2.3±0.9	−2.6±0.3	−4.9	−7.5	0.9
**7**	41±15	4.0±1.0	−9.9	−5.9	0.7

[a] *K*_d_, Δ*H*, and *N* (stoichiometry) are directly measured parameters, which are determined from the fit of binding data. The error is the fitting error, so it shows how good the fit (and corresponding binding equation) describes the experimental data. *N*<1 means that the real concentration of binding protein is lower than the total protein concentration in the sample (as determined by UV spectroscopy). The Gibbs free energy and entropic contribution to binding are estimated from the following equations: Δ*G*=*RT*ln(*K*_d_) and Δ*G*=Δ*H*−*T*Δ*S*.

### In vitro KDM4A inhibition assays

Ligands **1**–**5** were previously tested by us in a fragment‐based screening against the closely related isozyme KDM4A. In brief, ligand **1** was the most potent compound, with an IC_50_ value of 46.64±0.94 μm in a formaldehyde dehydrogenase (FDH)‐coupled assay. In the antibody‐based LANCE Ultra assay, the potency was as high as 2.38±0.37 μm. The two control compounds, **4** and **5**, in which one structural motif required for potent binding (hydrazide or tetrazole) is replaced or masked, exhibit no inhibition.^19^ The two derivatives **6** and **7**, with an aromatic spacer, were characterized as noninhibiting. Apparent IC_50_ values from both in vitro assays are reported in Table 4. The apparent potency is much higher in the FDH‐coupled assay than that in the LANCE Ultra assay, which is in contrast to previous observations for this and other structural classes of inhibitors.^21^ These results indicate an assay artifact in the FDH assay. Indeed, a counterscreening assay revealed that compounds **6** and **7** also negatively influenced the conversion of formaldehyde by FDH in a KDM4A‐free setup, potentially by directly inhibiting FDH, by reacting with released formaldehyde, or by quenching the fluorescence signal (see Figure S1 in the Supporting Information). As such, the results from the orthogonal LANCE Ultra assay appear to be more reliable; this means that compounds **6** and **7** are, at best, very weak KDM inhibitors. This is in agreement with the much less favorable binding properties observed in the ITC measurements (Table 3).

**Table 4:** Apparent in vitro potency of test compounds against isolated KDM4A in the FDH and LANCE assays.

Ligand		IC_50_ [μm]	
		FDH	LANCE Ultra
**6**		21.8	216±33
**7**		28.3	195±39

## Discussion

The aim of this work was to investigate the mode of binding of the histone demethylase KDM4D in complex with tetrazolylhydrazide compounds. Structural data describe, in detail, the interactions of this molecular scaffold with the KDM4 cofactor binding site and might be very helpful in the further development of inhibitors of the KDM4 subfamily. In our opinion, the most desired compounds should exhibit high affinity to make them potent binders and, at the same time, they should possess physicochemical properties that would allow them to penetrate cells across membranes. Only the combination of the two properties in one compound would identify a compound as one displaying high anti‐KDM4 activity in vivo. To the best of our knowledge, there is a limited number of KDM4 binding compounds, to date, that feature both properties13b and tetrazolylhydrazides might fill this gap.

### Role of the carboxyl group in KDM4 inhibitors

**Figure 4:**
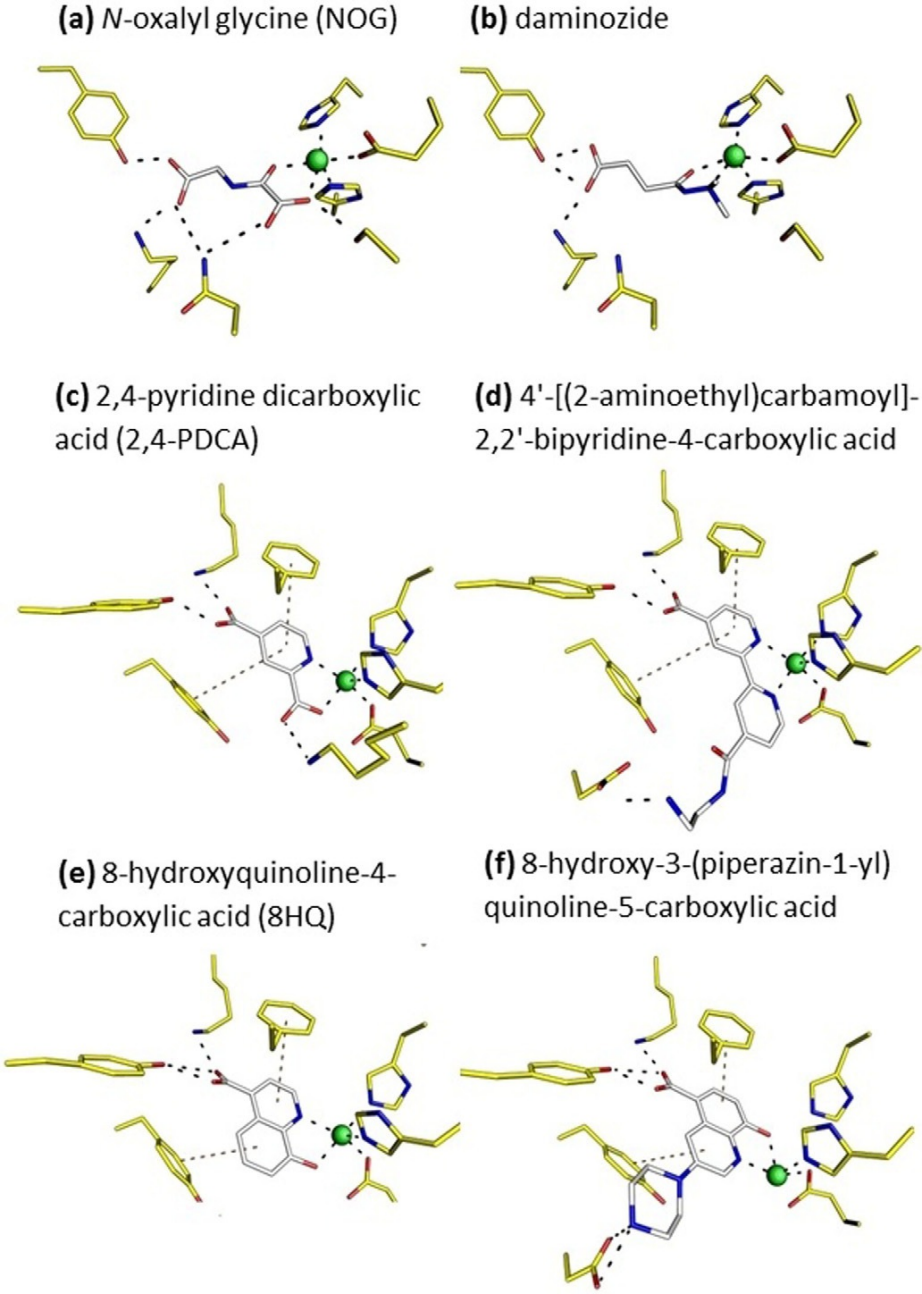
Interactions of various, previously described ligands containing carboxylic moieties with KDM4D (A, C) and KDM4A (B, D, E, F). Daminozide: *N*‐(dimethylamino)succinamic acid. Ligands are colored gray, active‐site residues are colored yellow, and the metal ions are shown as green spheres. Polar interactions are indicated as black dashed lines. The orange dashed lines highlight aromatic stacking interactions.

Early developed analogues of 2OG usually contain either one or two carboxyl groups, for example, *N*‐oxalylglycine (NOG; PDB ID: https://www.rcsb.org/structure/4D6S, unpublished) or 2,4‐pyridinedicarboxylic acid (2,4‐PDCA; PDB ID: https://www.rcsb.org/structure/4D6Q, unpublished; Figures 4 A, C). In all cases, one of the carboxylic groups is positioned in the same way as that in the naturally occurring cofactor, 2OG. Other scaffolds, which contain one carboxylic group, are, for instance, 2,2‐bipyridines13d (PDB ID: https://www.rcsb.org/structure/3PDQ), 8‐hydroxyquinoline (8HQ, PDB ID: https://www.rcsb.org/structure/4BIS), and derivatives of 8HQ13i (8‐hydroxy‐3‐(piperazin‐1‐yl)quinoline‐5‐carboxylic acid; PDB ID: https://www.rcsb.org/structure/3RVH; Figure 4 D–F). Several attempts were made to enhance the cell permeability of such KDM4 binding molecules. For instance, ester modifications were introduced to produce a prodrug, for example, in the case of MethylStat, which is a pan‐KDM inhibitor.^22^ However, it has been reported repeatedly that the carboxyl group was needed to maintain the potency of the inhibitor molecules. This can easily be explained by examining the interactions of the carboxyl groups with the enzyme, which are highlighted in several crystal structures of KDM4 complexes with ligands (Figure 4). Another strategy to eliminate carboxylate groups, different from a simple ester modification, is their replacement by isosteric groups. One such group is the tetrazole moiety. Given that, it seems surprising that no such structures of complexes have been reported for KDM4 proteins, to date, although tetrazolylhydrazide compounds in KDM4 inhibition were first reported in 2015 by Rüger et al.^19^

### Role of the hydrazide group in KDM4 inhibitors

Hydrazide‐containing ligands display a bidentate coordination of the metal ion. This is clearly evidenced by the structures of KDM4D in complex with compounds **1** to **3** and **5** to **7**. Substitution of the hydrazide group in compound **4** by a simple ethyl ester was not tolerated and resulted in a different mode of binding of compound **4** to KDM4D with respect to the aliphatic part of the molecule. It seems as if the unmodified hydrazide is necessary for binding to the active‐site metal ion, but also for interacting with neighboring active‐site residues, Ser200 and Asn202. Among all reported structures, to date, the nitrogen atom coordinating the metal in the same position as that in the hydrazide ligands described herein is observed only in daminozide, which is a compound described as a potential JmjC oxygenase inhibitor (PDB ID: https://www.rcsb.org/structure/4AI9).13p Closer inspection of the hydrogen‐bonding interactions clearly shows that the dimethylamino group in daminozide prevents binding to Ser200 and Asn202 (Figure 4 B). This interaction is observed in our compounds that exhibit low micromolar affinity: compounds **1** and **7**. The binding potency of the hydrazide is also shown in the case of compound **6**. The benzene ring is incorporated between the two main functional groups of the ligand; thus rigidifying it. Steric clashes prevent it from binding in the same bidentate manner as that reported for other tetrazolylhydrazide ligands and for 2OG. Nevertheless, it is still chelating the metal ion by replacing the water molecule for a hydrazide nitrogen atom. Consequently, the hydrazide appears to be a very powerful motif for chelating the metal ion in the active site of KDM4 enzymes.

### Tetrazole ring as a carboxyl group isostere

The second carboxyl group of the cofactor, which is distal to the chelated metal ion, was replaced by a tetrazole ring. It was anticipated that the tetrazole ring or its anionic counterpart, deprotonated tetrazolate, would occupy a similar position to that of the carboxylate group of 2OG and would thus maintain the interactions that were reported to have a great impact on binding affinity (Figure 4). Also, apart from the binding affinity, the hope was that, by introducing the tetrazole group, other properties would be introduced that would turn out to be advantageous for further development into drugs against KDM4 enzymes. Substitution of the carboxylic acid with the tetrazole moiety should make the compounds more lipophilic, and thus, improve cell permeability. It is clear that this notion remains speculative, until cell penetration experiments have been performed. However, it has been shown previously that this small, nitrogen‐rich functional heterocycle can serve simultaneously as an aromatic platform and as a functional interaction motif.^20^ Tetrazoles were reported as KDM4 binders by Rüger et al. in 2015.^19^ Among other reported KDM4 inhibitors, the most similar moiety to tetrazole is a triazole, which has been incorporated into a pyridine carboxylate scaffold. Many such derivatives have been synthesized and tested.13f However, their spatial position and orientation in the active site of the KDM4 enzymes is very different from that observed in the ligands reported herein. In contrast, tetrazole rings of ligands presented herein are never engaged in direct metal chelation, although an indirect, water‐mediated metal interaction could be observed for compound **4**. In most of the structures reported herein, the tetrazole ring is sandwiched between two aromatic residues in the active site, Phe189 and Tyr181. This position must be considered favorable based on the inspection of all of the structures. A proof of concept comes from the analysis of the structure of KDM4D in complex with compound **4**. Compound **4** does not have a hydrazide group chelating the metal ion, and thus, does not bind the metal directly. This lack of metal chelation explains why compound **4** cannot compete efficiently with the co‐substrate 2OG and does not inhibit KDM4. Nevertheless, its tetrazole ring is retained in the same position very close to those observed in the other tetrazole compounds **1** to **4** and **6** to **7**. It must therefore be concluded that this position is favorable for an aromatic platform that is able to engage in stacking interactions. The presence of two aromatic systems, for example, a tetrazole and a benzene group, for example, in compound **7** seems to increase the entropic contribution of the binding in KDM4D, but not in KDM4A. As a matter of fact, it is much higher than that in the case of ligand **1**. The position of the aromatic system, as reported herein, has not been observed previously, probably because most of the previously reported KDM4 inhibitors possess an aromatic ring as part of the metal chelating motif.

### Role of the active‐site residues of KDM4D

**Figure 5:**
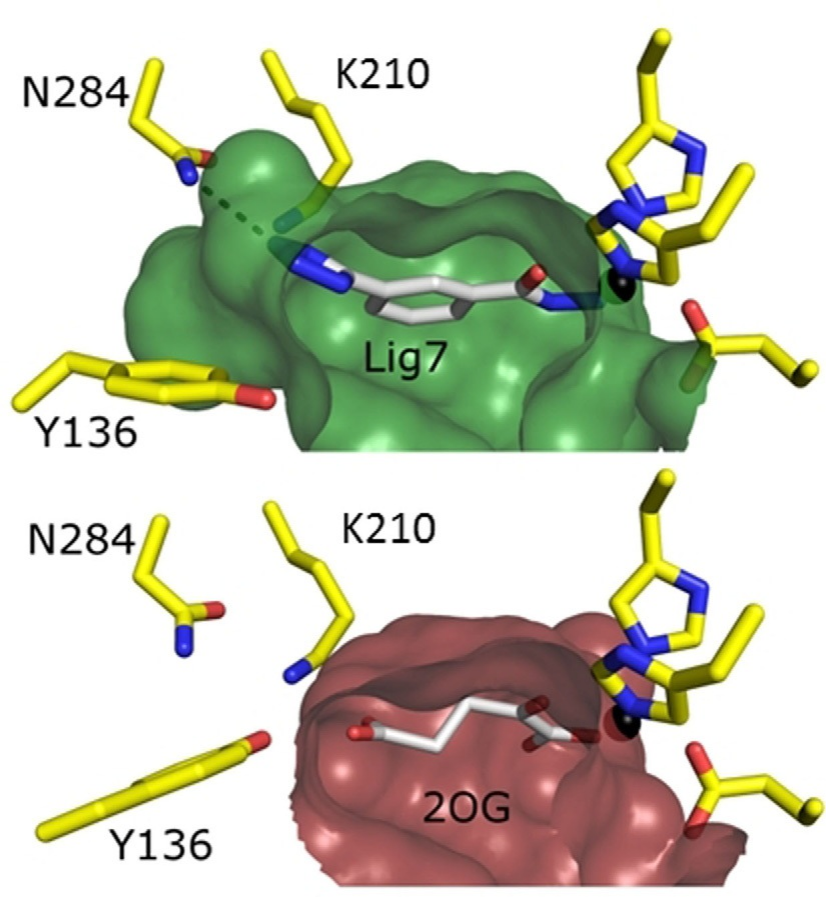
Surface representation of the binding pocket of ligand **7** colored in green. The 2OG binding cleft is shown in the bottom panel and colored in ruby. The active‐site residues are shown as yellow sticks. The unique polar interaction with Asn284 is shown as a dashed line. As a result of the movement of Tyr136, the 2OG binding cleft extends.

The obtained high‐resolution structures allow an investigation of the conformational changes of the amino acid residues in the active site upon ligand binding. Amino acids Lys210, Tyr136, Ser200, and Asn202 are all engaged in binding of the natural cofactor, 2OG. The positions and conformations of these residues are more or less the same in the cofactor‐bound and ‐free states of the enzyme. This suggests that the active site is in a ready state to accommodate the cofactor in a lock‐and‐key binding mode. The same position of these residues is also observed upon binding of compound **4** in the distal area of the active‐site cleft. In most instances, Asn202 points away from the metal center, except in the structure with compound **6**, in which the metal is chelated differently. A superposition of KDM4D structures in complex with tetrazolylhydrazides and a KDM structure in the presence of 2OG leads one to assume that binding of the tetrazolylhydrazide to the protein induces a restructuring of the active‐site residues. Asn202 is now in an “inward” position if the hydrazide group chelates the metal ion in the same bidentate manner as that in the natural cofactor binding, thanks to its internal nitrogen atom. This suggests an induced‐fit model for tetrazolylhydrazide binding to KDM4D. Interestingly, some of the compounds described herein induce the movement of Tyr136, which enlarges the binding cleft of the natural cofactor (Figure 5); thus making room for even more ligand atoms. It could be clearly observed in the complex structure with compound **7** that the tetrazole ring pushes Tyr136 away from Lys210 from a distance of 3.4 to 9.7 Å. At the same time, the tetrazole establishes a new interaction with Asn284. This residue is usually buried. It is a bit surprising, however, because similar interactions have been reported twice previously.13g They were observed in a pyrazolopyrimidine ligand structure (PDB ID: https://www.rcsb.org/structure/5KR7)13g and in its unmethylated form (PDB ID: https://www.rcsb.org/structure/5FJK). The ligand architecture that triggers movement of Tyr136 was reported to be essential for maintaining a high binding constant. However, the loss in the enthalpy contribution of ligand **7** compared with **1** is significant and might be due to losing the strong hydrogen bond with the hydroxy group of Tyr136. The mapping of flexible residues in the active side of KDM4D might be helpful in a process of rational design of higher affinity compounds.

## Conclusion

By describing a series of tetrazolylhydrazide compounds and by examining their respective binding modes to the target protein KDM4D, we have unveiled several unique interactions that were not observed previously and could potentially be useful in further inhibitor optimization. The tetrazole ring appears to be a suitable replacement for carboxyl groups. Because of its unique features, it can establish both aromatic and polar interactions. The hydrazide moiety seems to be a very powerful motif for metal chelation in KDM4 proteins. In contrast to other previously described compounds that target KDM4 proteins, the new compounds discussed herein might display more desirable physicochemical properties. The new scaffold fills an important gap in KDM4 inhibition and opens up a new path to developing compounds with high affinity and possibly increased permeability, at the same time. The tetrazolylhydrazide scaffold also presents several possibilities for ligand extension and branching. This would allow the creation of a ligand that could extend into other binding sites, for instance, the histone binding site, which has not yet been explored in the present series of compounds.

## Experimental Section

### Ligand synthesis

The synthesis of compounds **1**–**5** was described previously by Rüger et al.^19^

### Protein expression and purification

cDNA encoding the catalytic domain of human KDM4D, comprising residues 1–342, and KDM4A, comprising residues 1–359, was purchased from Source Bioscience (Nottingham, UK). It was cloned into the pQTEV expression vector, which encoded an N‐terminal hexahistidine tag. The proteins were purified by means of affinity chromatography. The binding buffer consisted of 50 mm HEPES, pH 7.5; 500 mm NaCl; 20 mm imidazole; and 1 mm tris(2‐carboxyethyl)phosphine (TCEP). Protein was recovered from resins by using elution buffer consisting of 50 mm HEPES, pH 7.5; 500 mm NaCl; 300 mm imidazole; and 1 mm TCEP. The His‐tagged protein was then processed by TEV protease to remove the N‐terminal affinity tag prior to further purification. In the final stage of purification, the protein sample was subjected to size‐exclusion chromatography in the following buffer: 10 mm HEPES, pH 7.5; 300 mm NaCl; 5 % (*w*/*v*) glycerol; and 0.5 mm TCEP.

### Protein crystallization

KDM4D crystals were grown by using the sitting‐drop vapor‐diffusion method at 291 K. The well solution consisted of 24 % (*w*/*v*) polyethylene glycol (PEG‐3350), 180 mm ammonium sulfate, and 0.1 m HEPES buffer at pH 7.0. The 19 mg mL^−1^ solution of protein consisted of 0.5 m NaCl, 5 % (*v*/*v*) glycerol, 1 mm TCEP, and 10 mm HEPES buffer at pH 7.5. A Gryphon crystallization robot (Art Robbins Instruments) was used to mix 0.4 μL of well solution with an equal volume of protein solution on a 96‐well low‐profile Intelli‐Plate (Art Robbins Instruments).

### Data collection, processing, structure determination, and refinement

For data collection, crystals were presoaked in well solution supplemented with 10 mm NiCl_2_ for 10 min. Subsequently, they were transferred to the ligand soaking solution, consisting of 100–150 mm ligand in well solution (10–15 % final DMSO concentration), and incubated from 30 min to 24 h. The crystals were then cryoprotected by quickly immersing them in well solution complemented with 20 % (*v*/*v*) ethylene glycol and then flash‐cooled in liquid nitrogen.^23^ XRD data were collected on beamlines BL14.1 and BL14.3 at the BESSY II electron storage ring operated by the Helmholtz‐Zentrum Berlin^24^ by using a PILATUS 6M and a Rayonix MX225 charge‐coupled device (CCD) detector, respectively. The data were integrated and scaled by using XDSAPP.^25^ All relevant data collection and processing statistics are given in Table 1. The structures were solved by simple molecular replacement by using a published KDM4 structure (PDB ID: https://www.rcsb.org/structure/4HON 13m) as a search model and the program Phaser.^26^ The resulting model was initially refined with REFMAC and subsequently by phenix.refine.^27^ Atomic displacement parameters (ADPs) were refined anisotropically for all protein atoms individually. The ligand occupancy values were determined by means of refinement by using phenix.refine from starting values of 0.75 in each case. The final model was obtained after several cycles of refinement and manual adjustments by using Coot^28^ and validated. All refined coordinate sets and the corresponding structure factor amplitudes have been deposited in the Protein Data Bank. All refinement statistics are presented in Table 2.

### KDM4A FDH assay

The FDH‐coupled demethylase activity assay was adapted from ref. 29 and performed in a total volume of 20 μL on white OptiPlate‐384 microtiter plates (PerkinElmer, Waltham, MA, USA) with 50 mm HEPES buffer at pH 7.50 containing 0.01 % Tween‐20. A solution of KDM4A 1–359 (0.10 mg mL^−1^, 2.4 μm) was preincubated with compound solutions of varying concentration (0–400 μm) in DMSO at room temperature for 10 min. A substrate solution containing 100 μm ascorbic acid, 10 μm FeSO_4_, 0.001 U μL^−1^ FDH, 500 μm NAD^+^, 50 μm 2OG, and 35 μm H3K9me3 substrate peptide ARK(me3)‐STGGK‐NH2 (Peptide Specialty Laboratories, Heidelberg, Germany) was added (final concentrations). The final DMSO concentration was 2 % in all wells. The fluorescence intensity of the product formed, NADH, was measured at *λ*_ex_=330 nm and *λ*_em_=460 nm on a POLARstar Optima microplate reader (BMG Labtech, Ortenberg, Germany) immediately after addition (*t*=0) and after 1 h incubation on a horizontal shaker at 37 °C. Values were blank‐corrected and the difference in intensity at *t*=1 and 0 h was taken as a measurement of enzyme activity. Activity, in percent, was compared with that of compound‐free DMSO control and no‐substrate negative control. Inhibition curves were analyzed by means of sigmoidal curve fitting by using GraphPad Prism 4.00 and IC_50_ values calculated from the fit parameters as mean± standard deviation (SD) from two independent experiments.

### KDM4A LANCE Ultra assay

The commercial antibody‐based LANCE Ultra demethylase activity assay (PerkinElmer, Waltham, MA, USA) was performed in a total volume of 10 μL on white OptiPlate‐384 microtiter plates (PerkinElmer) with 50 mm HEPES buffer at pH 7.50 containing 0.01 % Tween‐20 and 0.01 % BSA. A solution of 60 nm KDM4A 1–359 was preincubated with compound solutions of varying concentration (0–1000 μm) in DMSO at room temperature for 10 min. A substrate solution containing 100 μm ascorbic acid, 5 μm FeSO_4_, 1 μm 2OG, and 400 nm biotinylated H3K9me3 substrate peptide ARTKQTARK(me_3_)‐STGGKAPRKQLA‐GGK(biotin) (BPS Bioscience, San Diego, CA, USA) was added (final concentrations). The final DMSO concentration was 5 % in all wells. Plates were incubated on a horizontal shaker at room temperature for 45 min. Reactions were stopped by the addition of 10 μL of detection mix containing 2 nm europium‐labeled anti‐H3K9me_2_ LANCE antibody (PerkinElmer), 50 nm ULight‐streptavidin dye (PerkinElmer), and 1 mm EDTA in 1× LANCE detection buffer (PerkinElmer; final concentrations). Plates were again incubated on a horizontal shaker at room temperature for 60 min. FRET intensity was measured on an EnVision 2102 multilabel plate reader (PerkinElmer) at *λ*_ex_=340 nm and *λ*_em_=665 nm with a delay of 100 μs. Values were blank‐corrected and activity in percent was compared with compound‐free DMSO control and no‐enzyme negative control. Inhibition curves were analyzed by means of sigmoidal curve fitting with GraphPad Prism 4.00 and IC_50_ values calculated from the fit parameters as mean±SD from two independent experiments.

### FDH counterscreening assay

To evaluate the effect of test compounds on the FDH detection system, a modified assay was performed. The test was performed on white OptiPlate‐384 microtiter plates (PerkinElmer, Waltham, MA, USA) and the total test volume was 20 μL. The assay buffer was 50 mm HEPES at pH 7.50 and 0.01 % Tween‐20. Formaldehyde (40 μm) in assay buffer was preincubated for 10 min with compound solutions of varying concentration (10, 100, 400 μm) in DMSO at room temperature. A solution containing 100 μm ascorbic acid, 10 μm FeSO_4_, 0.001 U μL^−1^ FDH, 500 μm NAD^+^, and 50 μm 2OG was added. The final DMSO concentration was 2 % in all wells. The fluorescence intensity of the product NADH was measured at *λ*_ex_=330 nm and *λ*_em_=460 nm on a POLARstar Optima microplate reader (BMG Labtech, Ortenberg, Germany) immediately after addition (*t=*0) and after 1 h incubation on a horizontal shaker at 37 °C. Values were blank‐corrected and the difference in intensity at *t=*1 and 0 h was taken as a measurement of FDH activity. The activity, in percent, was given relative to that of the compound‐free DMSO control and no‐enzyme negative control.

### ITC measurements

ITC measurements on KDM4D and the respective tetrazolylhydrazide ligands were performed by using a MicroCal PEAQ‐ITC microcalorimeter (Malvern Panalytical GmbH, Germany). Experiments were performed in 10 mm HEPES buffer at pH 7.5, 0.5 m NaCl, 5 % (*v*/*v*) glycerol, and 1 mm TCEP at 20 °C. The protein concentration was determined spectrophotometrically at *λ*=280 nm by using the calculated molar extinction coefficient. The tetrazole ligands were diluted 100–250 times in buffer up to the working concentration from stock solutions in DMSO. The DMSO concentration in the resulting solutions ranged from 0.4 to 1 %. Solutions of KDM4D were supplemented with the corresponding amount of DMSO to avoid thermal effects as a result of buffer mismatch. Tetrazole ligands were titrated in 13 or 19 small steps (2–3 μL) into a solution of protein in the calorimeter cell. Other experimental settings included a spacing time of 180 s and a filtering period of 5 s. For each ligand, all measurements were performed two or three times. For all experiments, the instrument software (MicroCal PEAQ‐ITC Analysis) was used for baseline adjustment, peak integration, and normalization of the reaction heats, with respect to the molar amount of injected ligand, as well as for data fitting and binding parameter evaluation. The thermodynamic parameters for ligands showing measurable heat signals are presented in Table 3.

## Conflict of interest

*The authors declare no conflict of interest*.

## Supporting information

As a service to our authors and readers, this journal provides supporting information supplied by the authors. Such materials are peer reviewed and may be re‐organized for online delivery, but are not copy‐edited or typeset. Technical support issues arising from supporting information (other than missing files) should be addressed to the authors.

Supplementary
