# Supplementary material for: Structure‐Based Screening of Tetrazolylhydrazide Inhibitors versus KDM4 Histone Demethylases
Source: ChemMedChem. 2019 Oct 10;14(21):1828–39. doi: 10.1002/cmdc.201900441 (PMC6899576; doi:10.1002/cmdc.201900441)
Supplement: Supplementary file 1 — Supplementary [file CMDC-14-1828-s001.pdf]

CHEMMEDCHEM
Chemistry & Drug Discovery

Supporting Information

Structure-Based Screening of Tetrazolylhydrazide Inhibitors versus KDM4 Histone Demethylases

Piotr H. Małecki,[a, b, f] Nicole Rüger,[c] Martin Roatsch,[d, g] Oxana Krylova,[e] Andreas Link,[c] Manfred Jung,[d] Udo Heinemann,[b] and Manfred S. Weiss*[a]

cmdc_201900441_sm_miscellaneous_information.pdf

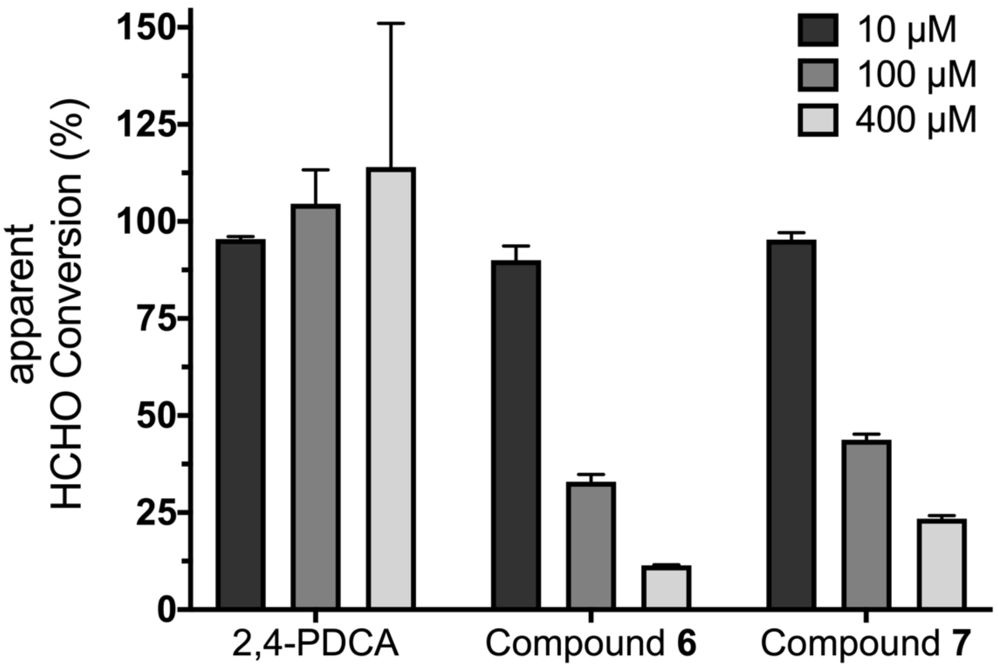

Supporting Figure 1: Results from an FDH-counterscreen assay to determine to which extent test compounds interfere with the FDH-based KDM activity detection. The FDH-coupled KDM4A assay was modified by replacing KDM enzyme with exogenously supplied formaldehyde, while keeping all other assay parameters constant. While the standard *in vitro* KDM inhibitor 2,4-PDCA had no effect on the apparent HCHO conversion by FDH, compounds **6** and **7** dramatically reduced the fluorescence signal, even though no KDM was present. Thus, the observed apparent KDM4A inhibition in this assay most likely stems from an assay artifact (e. g. fluorescence quenching, FDH inhibition, HCHO scavenging, etc). The orthogonal LANCE*Ultra* KDM inhibition assay, which does not rely on FDH detection, also revealed that **6** and **7** are only inhibiting in the range of 200 µM.
